# Comparative efficacy and safety of the left versus right radial approach for percutaneous coronary procedures: a meta-analysis including 6870 patients

**DOI:** 10.1590/1414-431X20154571

**Published:** 2015-06-23

**Authors:** S.L. Xia, X.B. Zhang, J.S. Zhou, X. Gao

**Affiliations:** Department of Cardiology, Affiliated Nanjing Jiangbei People's Hospital, Southeast University, Nanjing, Jiangsu, China

**Keywords:** Radial approach, Percutaneous coronary, Meta-analysis

## Abstract

The radial approach is widely used in the treatment of patients with coronary artery disease. We conducted a meta-analysis of published results on the efficacy and safety of the left and right radial approaches in patients undergoing percutaneous coronary procedures. A systematic search of reference databases was conducted, and data from 14 randomized controlled trials involving 6870 participants were analyzed. The left radial approach was associated with significant reductions in fluoroscopy time [standardized mean difference (SMD)=-0.14, 95% confidence interval (CI)=-0.19 to -0.09; P<0.00001] and contrast volume (SMD=-0.07, 95%CI=-0.12 to -0.02; P=0.009). There were no significant differences in rate of procedural failure of the left and the right radial approaches [risk ratios (RR)=0.98; 95%CI=0.77-1.25; P=0.88] or procedural time (SMD=-0.05, 95%CI=0.17-0.06; P=0.38). Tortuosity of the subclavian artery (RR=0.27, 95%CI=0.14-0.50; P<0.0001) was reported more frequently with the right radial approach. A greater number of catheters were used with the left than with the right radial approach (SMD=0.25, 95%CI=0.04-0.46; P=0.02). We conclude that the left radial approach is as safe as the right radial approach, and that the left radial approach should be recommended for use in percutaneous coronary procedures, especially in percutaneous coronary angiograms.

## Introduction

The radial approach has been demonstrated to be as effective as the femoral approach for both diagnostic and interventional coronary procedures (1,2). When compared with the femoral approach, the benefits of the radial approach include a lower incidence of entry site complications, decreased patient discomfort, reduced occurrence of cardiovascular events, and lower total variable procedural costs (3 4 5). The radial approach, which is safe and effective for diagnostic and interventional procedures, has been widely used in the treatment of patients with coronary artery disease (3). Since its first reported use for coronary angiography in 1989 (6), many researchers have published reports on the management of the radial artery approach (7). Most clinical studies have focused solely on the right radial approach (RRA) and rarely consider the left radial approach (LRA). Indeed, the RRA presents technical obstacles related to anatomy and clinical practice. For instance, a higher incidence of tortuosity of the subclavian artery and radial-ulnar artery loop are expected with the RRA as well as a longer learning curve (8 9 10 11 12). However, the RRA is still generally used in clinical practice despite its disadvantages.

Recent investigations found that the LRA was associated with lower fluoroscopy time (FT) and operator radiation exposure (13,14). Increased interest in the LRA, which offers all the advantages of the RRA and avoids most of its disadvantages, has led to reports proposing that the LRA offers greater benefits than the RRA (15,16). However, the evidence supporting these proposals is not robust, and recent randomized controlled trials (RCTs) have yielded conflicting results (13,). Previous meta-analyses of trials comparing procedural failures, procedural time, and fluoroscopy time concluded that the LRA was preferable to the RRA for diagnostic or interventional coronary procedures (18 19 20). However, some important variables and recent RCTs have not yet been considered.

In this meta-analysis of RCTs, we evaluated the efficacy and safety of the LRA compared with the RRA in patients undergoing percutaneous coronary procedures with regard to procedural failures, fluoroscopy time, procedural time, contrast volume, tortuosity of the subclavian artery, and the number of catheters used.

## Material and Methods

### Search strategy and study selection

We searched several reference databases, including PubMed, the Cochrane Central Register of Controlled Trials (CENTRAL), and the Web of Science, for listings up to October 2014 using different combinations of the following key words: left AND right AND radial AND (transradial OR coronary). Additional relevant articles were obtained by scanning conference summaries and reference lists. No language restrictions were applied.

RCTs of the LRA versus RRA for percutaneous coronary procedures were selected for analysis from among the retrieved publications. Trials enrolling patients who underwent a percutaneous coronary angiogram were included whether or not the procedure was followed by a coronary intervention during the study period. Results obtained from patients who had previous coronary artery bypass graft surgery were excluded.

The primary outcome measures were the proportion of procedural failures and the standardized mean difference (SMD) in changes of fluoroscopy time from baseline to endpoint. The secondary outcome measures were the SMD in changes of procedure time and contrast volume from baseline to endpoint. We also evaluated the proportion of procedures with tortuosity of the subclavian artery, and the number of catheters used. When the standard deviations (SD) of absolute changes from the baseline were not available from individual trials, they were imputed as described in the Cochrane Handbook (21).

### Data extraction and quality assessment

Two reviewers independently identified the articles to be analyzed by their inclusion and exclusion criteria, assessed their quality, and completed a standardized data extraction form. Any disagreements were resolved via discussion. The data abstracted included year of publication, study location, number of study patients, patient characteristics, and outcome data (procedure failure, fluoroscopy time, procedure time, contrast volume, tortuosity of the subclavian artery, and the number of catheters used during the procedure). The methodological quality of the studies was assessed using the Risk of Bias assessment tool from the Cochrane Handbook (21).

### Statistical analysis

We performed a pairwise meta-analysis using Review Manager (RevMan version 5.3, http://tech.cochrane.org/revman). We calculated the pooled estimates of the SMDs with 95% confidence intervals (CIs) for continuous outcomes and risk ratios (RRs) with 95%CIs for categorical outcomes between two direct comparisons (21). Heterogeneity of treatment effects across studies was assessed by I^2^ and the Cochrane Q test (21,22). I^2^ values of 25, 50, and 75% represented low, moderate, and high heterogeneity, respectively (23). With low heterogeneity for outcome data, a fixed-effects model was used. A random-effects model was used to analyze data with moderate or high heterogeneity. A P value of ≤0.05 was taken as statistically significant. Publication bias was examined by funnel plots. Sensitivity analysis was conducted to evaluate high heterogeneity. Separate subgroup analyses of fluoroscopy time, procedural time, and contrast volume for cardiac catheterization in angiography and percutaneous coronary intervention (PCI) studies were conducted.

## Results

**Figure 1:**
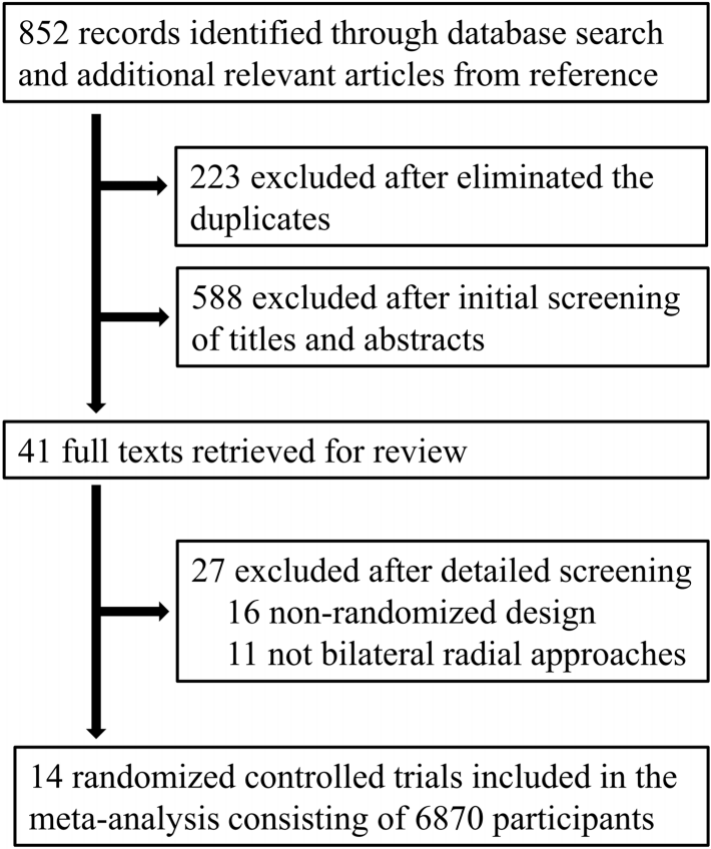
Flowchart of study selection.

As shown in Figure 1, we initially identified 852 records from key reference databases and additional relevant articles; 629 potentially relevant studies were kept after duplicates were eliminated. Of those, a total of 588 studies were excluded after a review of the abstracts revealed that they were either not relevant or not RCTs. An additional 27 of the remaining 41 studies were eliminated after a reading of the full text. A total of 14 RCTs involving 6870 participants satisfied the inclusion criteria and were included in this meta-analysis (13,1517,) .

Three of the 14 RCTs were from North America (27,28,30), 7 were from Europe (13,16,17,24,25,31,33), and 4 were from Asia (15,26,29,32). Table 1 lists the characteristics of the included trials. All were published between 2004 and 2014, the number of participants ranged from 40 to 1540, and the largest two were conducted by Sciahbasi et al. and Hu et al. (13,26). Most were single center studies. The mean age of participants was approximately 59.3 years, about two-thirds (64%) were male, and the inclusion and exclusion criteria of the participants were well described in all studies. Eight studies involved participants undergoing only angiography (13,15,16,24,26,), six involved participants undergoing PCI (13,17,25,27,29,33), and only one study did not clearly describe the reason for the procedure (28). Mean age, country, number of patients, and proportions of females and patients with hypercholesterolemia, hypertension, and diabetes are reported in Table 1.

**Table:**
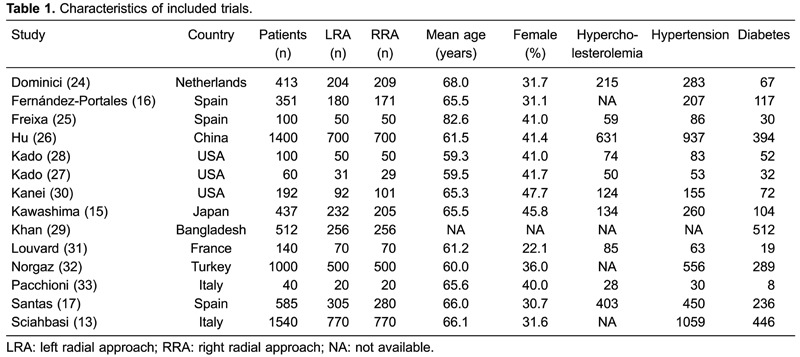

The overall quality of studies was rated as good, although many reports did not provide the methods of randomization, allocation concealment, or blinding. Outcomes with incomplete data were adequately described in all studies.

**Figure 2:**
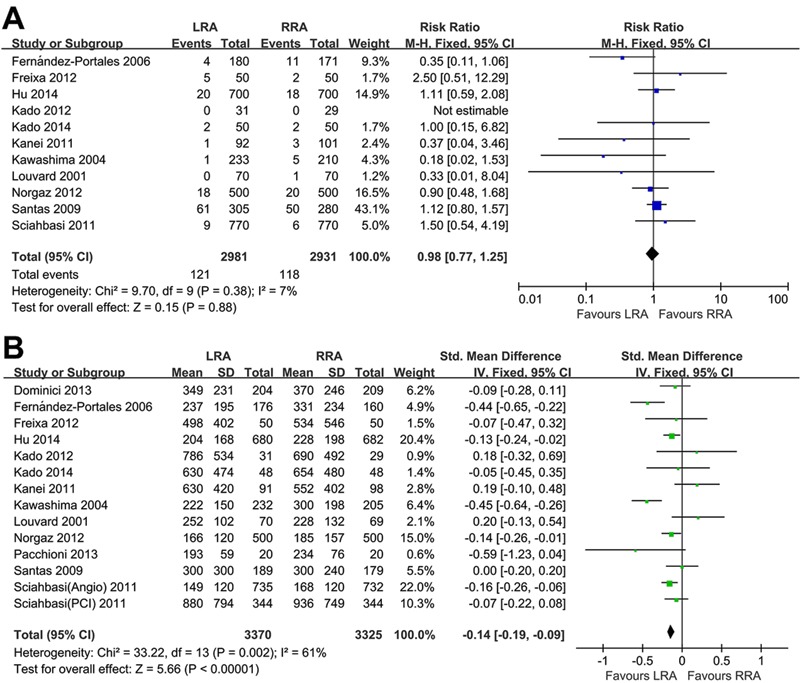
Meta-analyses of primary outcomes. *A*, Comparison of the left radial approach (LRA) versus the right radial approach (RRA) for procedural failure. *B*, Comparison of the LRA versus the RRA for fluoroscopy time. In the study of Sciahbasi (13), we reported the meta-analysis results of percutaneous coronary intervention (PCI) and diagnostic angiography (Angio). See Table 1 for numbers of all references cited.

Data on procedural failures that followed operations were available for 11 studies with 5912 participants (Figure 2A). There was no significant difference between the LRA and the RRA in the rate of procedural failure (RR=0.98, 95%CI=0.77-1.25). The LRA had a significantly shorter fluoroscopy time than the RRA (SMD=-0.14, 95%CI=-0.19 to -0.09; P<0.00001; Figure 2B).

**Figure 3:**
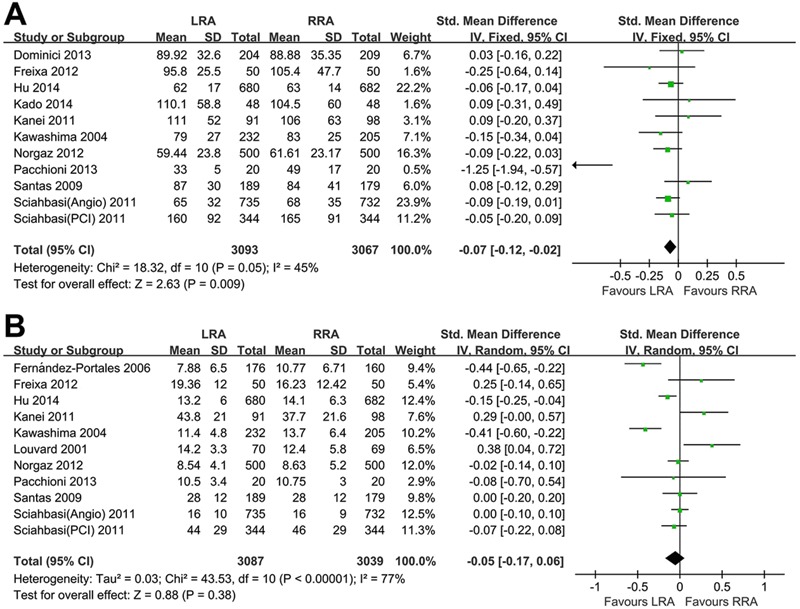
Meta-analysis of secondary outcomes. *A*, Comparison of the left radial approach (LRA) versus the right radial approach (RRA) for contrast volume. *B*, Comparison of the LRA versus the RRA for procedural time. In the study of Sciahbasi (13), we reported the meta-analysis results of percutaneous coronary intervention (PCI) and diagnostic angiography (Angio). See Table 1 for numbers of all references cited.

The secondary outcome of contrast volume showed a significant benefit for the LRA over the RRA in the study participants (SMD=-0.07, 95%CI=-0.12 to -0.02; P=0.009; Figure 3A). There was no significant difference between the two approaches in the duration of the procedure (SMD=-0.05, 95%CI=-0.17 to 0.06; P=0.38; Figure 3B). However, a significant, moderate heterogeneity was found among the studies reporting the duration of procedures (P<0.00001; I^2^=77%).

In addition, separate subgroup analyses of angiography versus PCI studies were conducted for fluoroscopy time, procedural time, and contrast volume. No significant difference in procedural time for LRA and RRA was found (SMD=-0.05, 95%CI=0.17-0.06; P=0.38; Supplementary Figure S1A-C), and the differences in fluoroscopy time and contrast volume were significant for angiography but not PCI studies.

**Figure 4:**
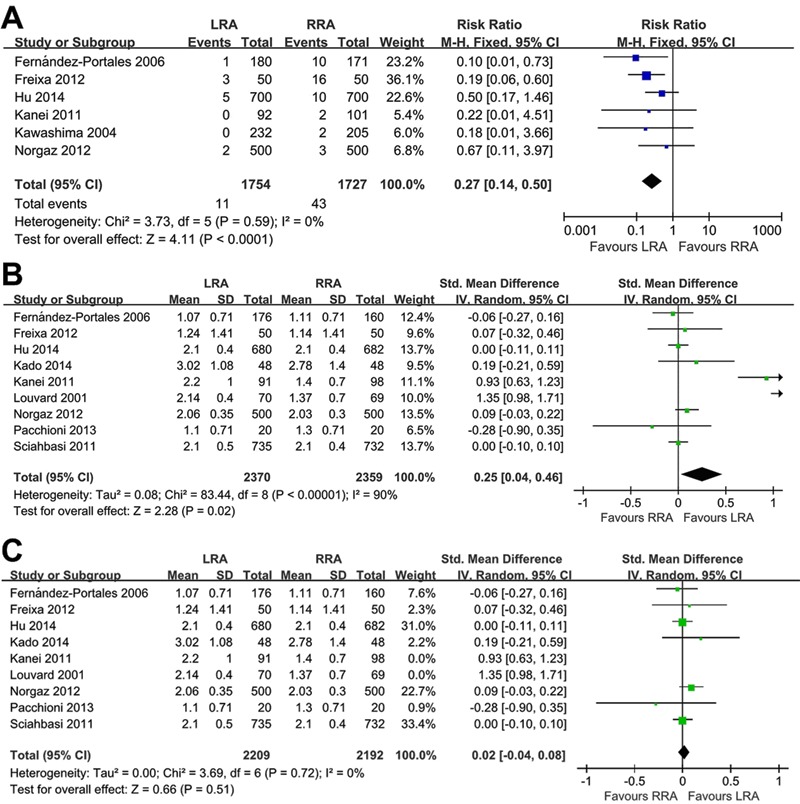
Meta-analysis of subclavian tortuosity and catheter count. *A*, Comparison of the left radial approach (LRA) versus the right radial approach (RRA) for tortuosity of the subclavian artery. *B*, Comparison of the LRA versus the RRA for catheter count. *C*, Sensitivity analysis of the catheter count meta-analysis excluding the studies by Kanei and Louvard (30,31). See Table 1 for numbers of all references cited.

**Figure 5:**
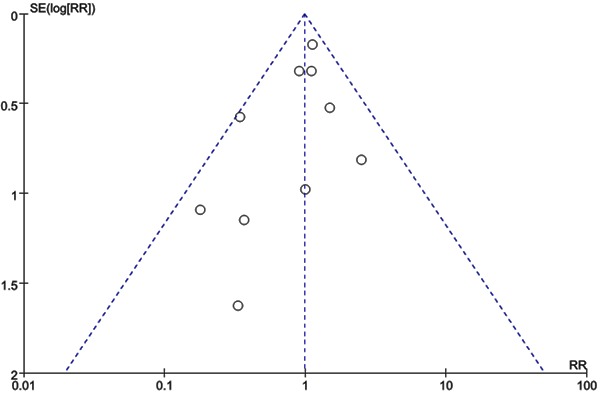
Funnel plot assessment of publication bias.

We also analyzed the available data on tortuosity of the subclavian artery (six studies) and the number of catheters used (nine studies). Tortuosity of the subclavian artery occurred significantly more often (RR=0.27, 95%CI=0.14-0.50; P<0.0001) in patients with the RRA (Figure 4A), and the number of catheters used was significantly greater in patients with the LRA (SMD=0.25, 95%CI=0.04-0.46; P=0.02; Figure 4B). High heterogeneity (P<0.00001; I^2^=90%) was found among the studies reporting the number of catheters used during the procedures, with the RRA using fewer catheters. However, after excluding the studies by Kanei et al. and Louvard et al. (30,31), a low heterogeneity was observed (P=0.72, I^2^=0%), and no significant difference was found in the test for overall effect (SMD=0.02; 95%CI=-0.04 to 0.08; P=0.51). Visual inspection of the funnel plot revealed no obvious publication bias (Figure 5).

## Discussion

In our literature review, we found recent studies comparing the LRA with the RRA, two of which were published in 2014 (26,28). We only included RCTs retrieved from key international databases to maintain the high quality and reliability of analysis. Moreover, data concerning subclavian tortuosity and catheter count were extracted to evaluate both radial approaches comprehensively. Meta-analysis of all 14 RCTs found that the LRA is effective for percutaneous coronary procedures and superior to the RRA in fluoroscopy time and contrast volume. In addition, based on the procedural failure results, the LRA is as safe as the RRA, with no differences found in procedural time. Subclavian tortuosity was much less frequent when the LRA rather than the RRA was used. However, the LRA used a greater number of catheters. The subgroup analysis revealed significant differences in the fluoroscopy time and contrast volume reported in the angiography and the PCI studies.

Analysis of procedural failure showed that the LRA was as safe as the RRA. Indeed, transradial approaches can be performed with a high success rate, low complication rate, and good angiographic quality (31). Furthermore, compared with the transfemoral approach, the transradial approaches show lower incidences of access-site bleeding complications, decreased patient discomfort, increased patient ambulation, and reduced hospital stays (34). Indeed, the analysis found that LRA is as valid an alternative to the femoral approach as the RRA (17).

The primary outcome of fluoroscopy time was found to be significantly longer for the RRA than the LRA, which may be attributable to right subclavian tortuosity that impeded the procedure. Kawashima et al. pointed out that improvements in catheters and X-ray systems might be expected to shorten fluoroscopy time and to decrease the amount of contrast material in both approaches (15).

Although no significant difference was found in the procedural time of the two approaches, the LRA still had a slight advantage. A longer procedural time with the RRA may be partly attributable to right subclavian tortuosity. However, the RRA, regardless of operator experience, is a more complex procedure to perform and thus cannot be resolved simply by overcoming the initial learning curve (16). In addition, the evidence reveals that patients older than 70 years of age who were treated with the RRA had a six-fold greater risk of prolongation of procedural time than those younger than 70 years of age (16). Moreover, longer procedural times and catheter manipulation in or around the neck vessels have been associated with increased risk of both silent and symptomatic stroke (35,36). Consequently, special attention is required when employing the RRA. In terms of contrast volume, the LRA showed a slight benefit compared with the RRA. More contrast was needed because more digital acquisitions are required when using the RRA, which also results in an increased cost of contrast materials (31).

The subclavian tortuosity results indicated that the LRA was superior to the RRA. Indeed, the radial artery is a small vessel that often shows spasms after percutaneous coronary procedures are completed. The presence of right subclavian artery-common brachiocephalic trunk (CBT) and CBT-aorta bifurcations encountered during the RRA always reduces the procedural success rate and increases the incidence of procedural failure (37,38). In contrast, the left subclavian artery is similar to the femoral artery, both of which stem directly from the aortic arch, thereby reducing the presence of tortuosity in the LRA (24).

In the catheter count analysis, a significant, high heterogeneity was found in the number of catheters used during the procedures (P<0.00001; I^2^=90%). When we conducted a sensitivity analysis by excluding the studies by Kanei et al. (30) and Louvard et al. (31), a low heterogeneity was observed (P=0.72, I^2^=0%), and no significant difference was found in the test for overall effect (SMD=0.02, 95%CI=-0.04 to 0.08; P=0.51), indicating that there was no significant difference between the LRA and the RRA. We considered that several operator and patient factors might have caused the high heterogeneity. First, the choice of catheters in the Kanei study was dependent on the discretion of the operator (30), which may have influenced the numbers that were used in that study. Second, the position of the operator was also important. In the Louvard study (31), the operator stood on the patient's left side whereas the operator in other studies stood on the right side (15,24). That may have influenced the number of catheters used based on the level of operator comfort during the procedure.

In the subgroup analyses, the fluoroscopy time and contrast volume in the LRA and RRA were significantly different only in angiography procedures, indicating that the advantages of the LRA might apply to percutaneous coronary angiograms but not to coronary interventions. A previous meta-analysis of studies comparing the two radial approaches came to the same conclusion (20).

In the past decade, an increasing number of studies have focused on establishing optimal access to conduct percutaneous coronary procedures, but few of these studies directly compared the operator radiation exposure among the different approaches. In the past year, two RCTs reported details about continuous operator radiation exposure (24,28). Both studies reported that the LRA was associated with a lower radiation dose absorbed by the operator, and was as effective as the RRA. The reduction of radiation exposure was similar in experienced and inexperienced operators. However, the operators felt uncomfortable during vascular access with the LRA in obese patients (28). Thus, increased operator discomfort may be proposed as a reason for not performing the LRA.

This meta-analysis had several limitations. First, although we included 14 high-quality RCTs for the meta-analysis, most of them were small-to-moderate size, single-center studies. Second, some results exhibited significant moderate heterogeneity. Third, data on dose-area products were not frequently reported in the included RCTs, so we were not able to assess this variable. Fourth, most of the included RCTs did not measure the absorbed radiation dose for either the patients or the operators. Fifth, the selection of catheters and the experience level of the operators were not consistent among the included RCTs. Despite the shortcomings of this meta-analysis, some of which may be unavoidable, we collected sufficient data from the included RCTs to evaluate the efficacy and safety of both transradial approaches for patients undergoing percutaneous coronary procedures.

In conclusion, the LRA is as safe as the RRA for percutaneous coronary procedures. The results showed significant benefits in using the LRA compared with the RRA, including reduced fluoroscopy time, contrast volume, and subclavian tortuosity. The results of this analysis support the LRA as the more prudent choice and the one that should be recommended for use in percutaneous coronary procedures, especially percutaneous coronary angiograms.

## Supplementary Material



## References

[1] Jolly SS, Yusuf S, Cairns J, Niemela K, Xavier D, Widimsky P (2011). Radial versus femoral access for coronary angiography
and intervention in patients with acute coronary syndromes (RIVAL): a randomised,
parallel group, multicentre trial. Lancet.

[2] Doyle BJ, Rihal CS, Gastineau DA, Holmes DR (2009). Bleeding, blood transfusion, and increased mortality
after percutaneous coronary intervention: implications for contemporary
practice. J Am Coll Cardiol.

[3] Agostoni P, Biondi-Zoccai GG, de Benedictis ML, Rigattieri S, Turri M, Anselmi M (2004). Radial versus femoral approach for percutaneous coronary
diagnostic and interventional procedures; Systematic overview and meta-analysis of
randomized trials. J Am Coll Cardiol.

[4] Jolly SS, Amlani S, Hamon M, Yusuf S, Mehta SR (2009). Radial versus femoral access for coronary angiography or
intervention and the impact on major bleeding and ischemic events: a systematic
review and meta-analysis of randomized trials. Am Heart J.

[5] Roussanov O, Wilson SJ, Henley K, Estacio G, Hill J, Dogan B (2007). Cost-effectiveness of the radial versus femoral artery
approach to diagnostic cardiac catheterization. J Invasive Cardiol.

[6] Campeau L (1989). Percutaneous radial artery approach for coronary
angiography. Cathet Cardiovasc Diagn.

[7] Kiemeneij F, Laarman GJ, Odekerken D, Slagboom T, van der Wieken R (1997). A randomized comparison of percutaneous transluminal
coronary angioplasty by the radial, brachial and femoral approaches: the access
study. J Am Coll Cardiol.

[8] Looi JL, Cave A, El-Jack S (2011). Learning curve in transradial coronary
angiography. Am J Cardiol.

[9] Goldberg SL, Renslo R, Sinow R, French WJ (1998). Learning curve in the use of the radial artery as
vascular access in the performance of percutaneous transluminal coronary
angioplasty. Cathet Cardiovasc Diagn.

[10] Yokoyama N, Takeshita S, Ochiai M, Koyama Y, Hoshino S, Isshiki T (2000). Anatomic variations of the radial artery in patients
undergoing transradial coronary intervention. Catheter Cardiovasc Interv.

[11] Burzotta F, Trani C, De Vita M, Crea F (2010). A new operative classification of both anatomic vascular
variants and physiopathologic conditions affecting transradial cardiovascular
procedures. Int J Cardiol.

[12] Dehghani P, Mohammad A, Bajaj R, Hong T, Suen CM, Sharieff W (2009). Mechanism and predictors of failed transradial approach
for percutaneous coronary interventions. JACC Cardiovasc Interv.

[13] Sciahbasi A, Romagnoli E, Burzotta F, Trani C, Sarandrea A, Summaria F (2011). Transradial approach (left vs right) and procedural
times during percutaneous coronary procedures: TALENT study. Am Heart J.

[14] Sciahbasi A, Romagnoli E, Trani C, Burzotta F, Sarandrea A, Summaria F (2011). Operator radiation exposure during percutaneous coronary
procedures through the left or right radial approach: the TALENT dosimetric
substudy. Circ Cardiovasc Interv.

[15] Kawashima O, Endoh N, Terashima M, Ito Y, Abe S, Ootomo T (2004). Effectiveness of right or left radial approach for
coronary angiography. Catheter Cardiovasc Interv.

[16] Fernandez-Portales J, Valdesuso R, Carreras R, Jimenez-Candil J, Serrador A, Romani S (2006). [Right versus left radial artery approach for coronary
angiography. Differences observed and the learning curve]. Rev Esp Cardiol.

[17] Santas E, Bodi V, Sanchis J, Nunez J, Mainar L, Minana G (2009). The left radial approach in daily practice. A randomized
study comparing femoral and right and left radial approaches. Rev Esp Cardiol.

[18] Guo X, Ding J, Qi Y, Jia N, Chu S, Lin J (2013). Left radial access is preferable to right radial access
for the diagnostic or interventional coronary procedures: a meta-analysis
involving 22 randomized clinical trials and 10287 patients. PLoS One.

[19] Biondi-Zoccai G, Sciahbasi A, Bodi V, Fernandez-Portales J, Kanei Y, Romagnoli E (2013). Right versus left radial artery access for coronary
procedures: an international collaborative systematic review and meta-analysis
including 5 randomized trials and 3210 patients. Int J Cardiol.

[20] De Rosa S, Torella D, Caiazzo G, Giampa S, Indolfi C (2014). Left radial access for percutaneous coronary procedures:
from neglected to performer? A meta-analysis of 14 studies including 7,603
procedures. Int J Cardiol.

[21] Higgins JPT, Green S (2011). Cochrane handbook for systematic reviews of
interventions .Version 5.1.0 (updated March 2011). The Cochrane Collaboration.

[22] Cochran W (1950). The comparison of percentages in matched
samples. Biometrika.

[23] Higgins JP, Thompson SG, Deeks JJ, Altman DG (2003). Measuring inconsistency in meta-analyses. BMJ.

[24] Dominici M, Diletti R, Milici C, Bock C, Placanica A, D'Alessandro G (2013). Operator exposure to x-ray in left and right radial
access during percutaneous coronary procedures: OPERA randomised
study. Heart.

[25] Freixa X, Trilla M, Feldman M, Jimenez M, Betriu A, Masotti M (2012). Right versus left transradial approach for coronary
catheterization in octogenarian patients. Catheter Cardiovasc Interv.

[26] Hu H, Fu Q, Chen W, Wang D, Hua X, Chen B (2014). A prospective randomized comparison of left and right
radial approach for percutaneous coronary angiography in Asian
populations. Clin Interv Aging.

[27] Kado H, Patel A, Suryadevara S, Angiolillo D, Box L, Zenni M (2012). Radial access: is there an increased risk of operator
radiation exposure during a right versus left radial approach?. J Am Coll Cardiol.

[28] Kado H, Patel AM, Suryadevara S, Zenni MM, Box LC, Angiolillo DJ (2014). Operator radiation exposure and physical discomfort
during a right versus left radial approach for coronary interventions: a
randomized evaluation. JACC Cardiovasc Interv.

[29] Kahn S, Kabir S (2012). Coronary procedures by left versus right transradial
approach in diabetic population. J Am Coll Cardiol.

[30] Kanei Y, Nakra NC, Liou M, Vales LL, Gowda R, Rosero H (2011). Randomized comparison of transradial coronary
angiography via right or left radial artery approaches. Am J Cardiol.

[31] Louvard Y, Lefevre T, Allain A, Morice M (2001). Coronary angiography through the radial or the femoral
approach: The CARAFE study. Catheter Cardiovasc Interv.

[32] Norgaz T, Gorgulu S, Dagdelen S (2012). A randomized study comparing the effectiveness of right
and left radial approach for coronary angiography. Catheter Cardiovasc Interv.

[33] Pacchioni A, Versaci F, Mugnolo A, Penzo C, Nikas D, Sacca S (2013). Risk of brain injury during diagnostic coronary
angiography: comparison between right and left radial approach. Int J Cardiol.

[34] Agostoni P, Biondi-Zoccai GG, de Benedictis ML, Rigattieri S, Turri M, Anselmi M (2004). Radial versus femoral approach for percutaneous coronary
diagnostic and interventional procedures; Systematic overview and meta-analysis of
randomized trials. J Am Coll Cardiol.

[35] Karalis DG, Quinn V, Victor MF, Ross JJ, Polansky M, Spratt KA (1996). Risk of catheter-related emboli in patients with
atherosclerotic debris in the thoracic aorta. Am Heart J.

[36] Busing KA, Schulte-Sasse C, Fluchter S, Suselbeck T, Haase KK, Neff W (2005). Cerebral infarction: incidence and risk factors after
diagnostic and interventional cardiac catheterization--prospective evaluation at
diffusion-weighted MR imaging. Radiology.

[37] Valsecchi O, Vassileva A, Musumeci G, Rossini R, Tespili M, Guagliumi G (2006). Failure of transradial approach during coronary
interventions: anatomic considerations. Catheter Cardiovasc Interv.

[38] Yoo BS, Yoon J, Ko JY, Kim JY, Lee SH, Hwang SO (2005). Anatomical consideration of the radial artery for
transradial coronary procedures: arterial diameter, branching anomaly and vessel
tortuosity. Int J Cardiol.

