# Supplementary material for: Comparative efficacy and safety of the left versus right radial approach for percutaneous coronary procedures: a meta-analysis including 6870 patients
Source: Braz J Med Biol Res. 2015 Jun 23;48(8):743–50. doi: 10.1590/1414-431X20154571 (PMC4541695; doi:10.1590/1414-431X20154571)

Figure S1. *A*, Subgroup analyses of angiography studies versus percutaneous coronary intervention (PCI) studies. Comparison of the left radial approach (LRA) versus the right radial approach (RRA) for fluoroscopy time. *B*, Subgroup analyses of angiography studies versus PCI studies. Comparison of the LRA versus the RRA for contrast volume. *C*, Subgroup analyses of angiography studies versus PCI studies. Comparison of the LRA versus the RRA for procedural time. See Table 1 of the article for numbers of all references cited.

A

	LRA			RRA				Std. Mean Difference
**Study or Subgroup**	**Mean**	**SD**	**Total**	**Mean**	**SD**	**Total**	**Weight**	**IV, Random, 95% CI**
**2.1.1 Angiography studies**								
Dominici 2013	349	231	204	370	246	209	8.5%	-0.09 [-0.28, 0.11]
Fernández-Portales 2006	237	195	176	331	234	160	7.7%	-0.44 [-0.65, -0.22]
Hu 2014	204	168	680	228	198	682	11.9%	-0.13 [-0.24, -0.02]
Kanei 2011	630	420	91	552	402	98	5.7%	0.19 [-0.10, 0.48]
Kawashima 2004	222	150	232	300	198	205	8.6%	-0.45 [-0.64, -0.26]
Louvard 2001	252	102	70	228	132	69	4.7%	0.20 [-0.13, 0.54]
Norgaz 2012	166	120	500	185	157	500	11.2%	-0.14 [-0.26, -0.01]
Sciahbasi(Angio) 2011	149	120	735	168	120	732	12.0%	-0.16 [-0.26, -0.06]
**Subtotal (95% CI)**			**2688**			**2655**	**70.2%**	**-0.15 [-0.27, -0.04]**
Heterogeneity: Tau² = 0.02; Chi² = 26.29, df = 7 (P = 0.0004); I² = 73%								
Test for overall effect: Z = 2.61 (P = 0.009)								
**2.1.2 PCI studies**								
Freixa 2012	498	402	50	534	546	50	3.7%	-0.07 [-0.47, 0.32]
Kado 2012	786	534	31	690	492	29	2.5%	0.18 [-0.32, 0.69]
Kado 2014	630	474	48	654	480	48	3.6%	-0.05 [-0.45, 0.35]
Pacchioni 2013	193	59	20	234	76	20	1.7%	-0.59 [-1.23, 0.04]
Santas 2009	300	300	189	300	240	179	8.1%	0.00 [-0.20, 0.20]
Sciahbasi(PCI) 2011	880	794	344	936	749	344	10.1%	-0.07 [-0.22, 0.08]
**Subtotal (95% CI)**			**682**			**670**	**29.8%**	**-0.05 [-0.16, 0.05]**
Heterogeneity: Tau² = 0.00; Chi² = 3.93, df = 5 (P = 0.56); I² = 0%								
Test for overall effect: Z = 1.00 (P = 0.32)								
**Total (95% CI)**			**3370**			**3325**	**100.0%**	**-0.13 [-0.21, -0.04]**
Heterogeneity: Tau² = 0.01; Chi² = 33.22, df = 13 (P = 0.002); I² = 61%								
Test for overall effect: Z = 2.81 (P = 0.005)								
Test for subgroup differences: Chi² = 1.51, df = 1 (P = 0.22), I² = 33.7%								

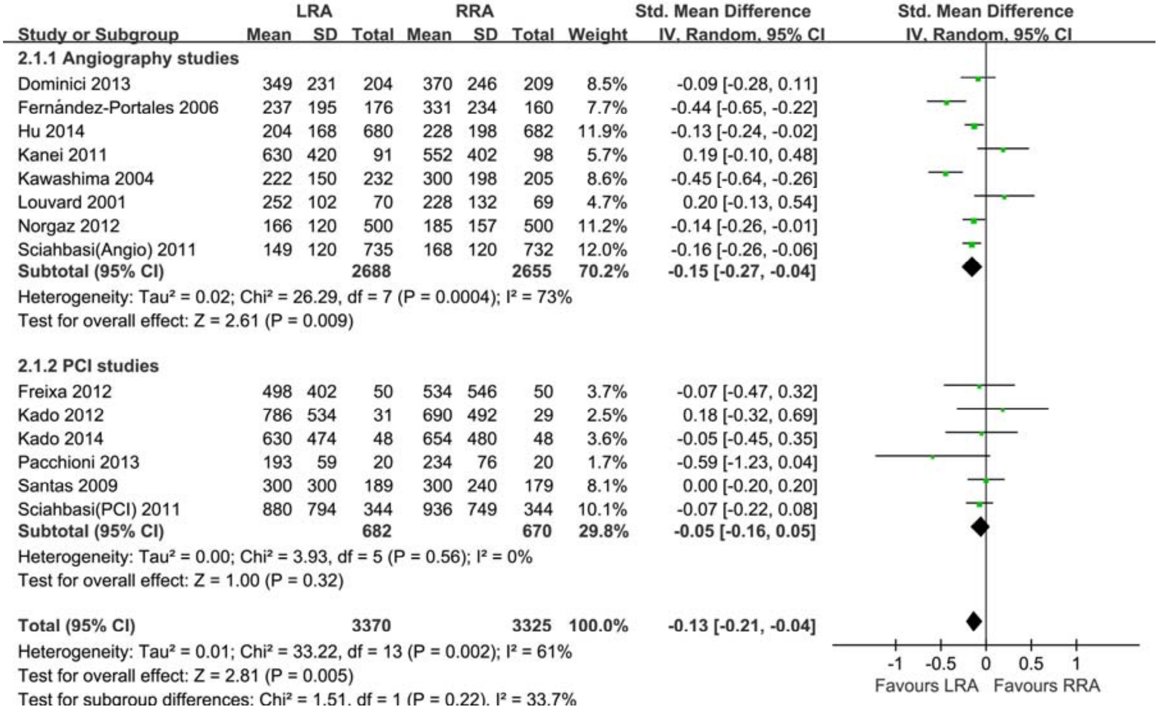

B

	LRA			RRA				Std. Mean Difference
**Study or Subgroup**	**Mean**	**SD**	**Total**	**Mean**	**SD**	**Total**	**Weight**	**IV, Random, 95% CI**
**2.3.1 Angiography studies**								
Dominici 2013	89.92	32.6	204	88.88	35.35	209	9.4%	0.03 [-0.16, 0.22]
Hu 2014	62	17	680	63	14	682	16.1%	-0.06 [-0.17, 0.04]
Kanei 2011	111	52	91	106	63	98	5.5%	0.09 [-0.20, 0.37]
Kawashima 2004	79	27	232	83	25	205	9.6%	-0.15 [-0.34, 0.04]
Norgaz 2012	59.44	23.8	500	61.61	23.17	500	14.4%	-0.09 [-0.22, 0.03]
Sciahbasi(Angio) 2011	65	32	735	68	35	732	16.5%	-0.09 [-0.19, 0.01]
**Subtotal (95% CI)**			**2442**			**2426**	**71.4%**	**-0.07 [-0.13, -0.02]**
Heterogeneity: Tau² = 0.00; Chi² = 3.21, df = 5 (P = 0.67); I² = 0%								
Test for overall effect: Z = 2.50 (P = 0.01)								
**2.3.2 PCI studies**								
Freixa 2012	95.8	25.5	50	105.4	47.7	50	3.2%	-0.25 [-0.64, 0.14]
Kado 2014	110.1	58.8	48	104.5	60	48	3.1%	0.09 [-0.31, 0.49]
Pacchioni 2013	33	5	20	49	17	20	1.2%	-1.25 [-1.94, -0.57]
Santas 2009	87	30	189	84	41	179	8.7%	0.08 [-0.12, 0.29]
Sciahbasi(PCI) 2011	160	92	344	165	91	344	12.3%	-0.05 [-0.20, 0.09]
**Subtotal (95% CI)**			**651**			**641**	**28.6%**	**-0.14 [-0.40, 0.11]**
Heterogeneity: Tau² = 0.06; Chi² = 14.98, df = 4 (P = 0.005); I² = 73%								
Test for overall effect: Z = 1.10 (P = 0.27)								
**Total (95% CI)**			**3093**			**3067**	**100.0%**	**-0.06 [-0.14, 0.01]**
Heterogeneity: Tau² = 0.01; Chi² = 18.32, df = 10 (P = 0.05); I² = 45%								
Test for overall effect: Z = 1.67 (P = 0.09)								
Test for subgroup differences: Chi² = 0.29, df = 1 (P = 0.59), I² = 0%								

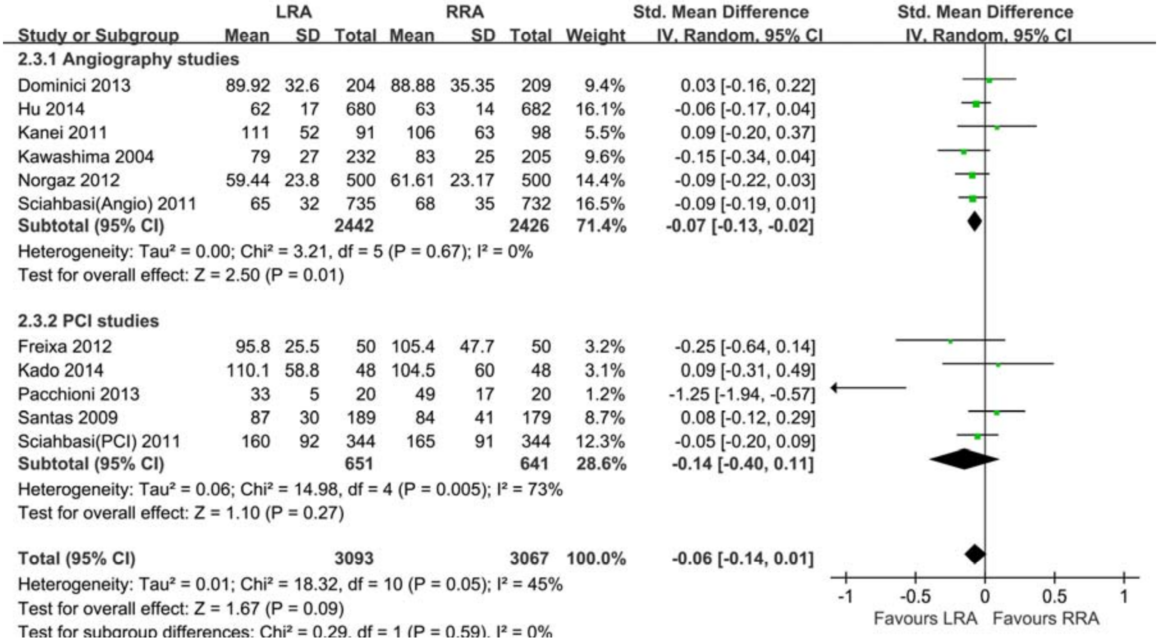

C

Study or Subgroup	LRA Mean	LRA SD	LRA Total	RRA Mean	RRA SD	RRA Total	Weight	Std. Mean Difference IV, Random, 95% CI
**2.2.1 Angiography studies**								
Fernández-Portales 2006	7.88	6.5	176	10.77	6.71	160	9.4%	-0.44 [-0.65, -0.22]
Hu 2014	13.2	6	680	14.1	6.3	682	12.4%	-0.15 [-0.25, -0.04]
Kanei 2011	43.8	21	91	37.7	21.6	98	7.6%	0.29 [-0.00, 0.57]
Kawashima 2004	11.4	4.8	232	13.7	6.4	205	10.2%	-0.41 [-0.60, -0.22]
Louvard 2001	14.2	3.3	70	12.4	5.8	69	6.5%	0.38 [0.04, 0.72]
Norgaz 2012	8.54	4.1	500	8.63	5.2	500	12.0%	-0.02 [-0.14, 0.10]
Sciahbasi(Angio) 2011	16	10	735	16	9	732	12.5%	0.00 [-0.10, 0.10]
**Subtotal (95% CI)**			**2484**			**2446**	**70.6%**	**-0.08 [-0.23, 0.08]**
Heterogeneity: Tau² = 0.04; Chi² = 40.12, df = 6 (P < 0.00001); I² = 85%								
Test for overall effect: Z = 0.94 (P = 0.35)								
**2.2.2 PCI studies**								
Freixa 2012	19.36	12	50	16.23	12.42	50	5.4%	0.25 [-0.14, 0.65]
Pacchioni 2013	10.5	3.4	20	10.75	3	20	2.9%	-0.08 [-0.70, 0.54]
Santas 2009	28	12	189	28	12	179	9.8%	0.00 [-0.20, 0.20]
Sciahbasi(PCI) 2011	44	29	344	46	29	344	11.3%	-0.07 [-0.22, 0.08]
**Subtotal (95% CI)**			**603**			**593**	**29.4%**	**-0.02 [-0.13, 0.09]**
Heterogeneity: Tau² = 0.00; Chi² = 2.34, df = 3 (P = 0.50); I² = 0%								
Test for overall effect: Z = 0.36 (P = 0.72)								
**Total (95% CI)**			**3087**			**3039**	**100.0%**	**-0.05 [-0.17, 0.06]**
Heterogeneity: Tau² = 0.03; Chi² = 43.53, df = 10 (P < 0.00001); I² = 77%								
Test for overall effect: Z = 0.88 (P = 0.38)								
Test for subgroup differences: Chi² = 0.30, df = 1 (P = 0.58), I² = 0%								

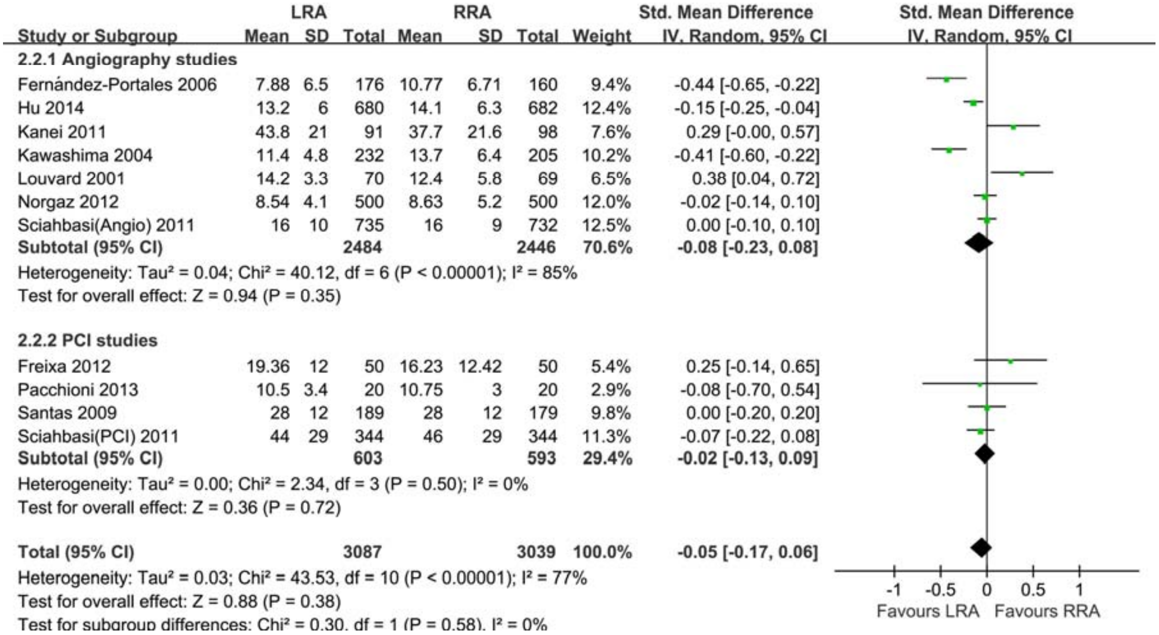

Supplement: Supplementary file 1 [file 1414-431X-bjmbr-48-08-00743-suppl001.pdf]
